# Clinical Anatomy and Assessment of the Elbow

**DOI:** 10.2174/1874325001711011347

**Published:** 2017-11-30

**Authors:** Alexander L Aquilina, Andrew J Grazette

**Affiliations:** University Hospitals Coventry and Warwickshire, Clifford Bridge Road, CV2 2DX, Coventry, UK

**Keywords:** Elbow, Clinical Anatomy, Assessment, Anatomy, Joint

## Abstract

**Background:**

The elbow is a complex synovial hinge joint comprising of three articulations. Satisfactory function and stability are provided by bony and soft tissue stabilising structures. Injuries around the elbow joint are common.

**Methods:**

A literature search was performed and the authors’ personal experiences reported.

**Results:**

The article discusses the osseous and ligamentous anatomy around the elbow joint and their relevance when assessing and managing elbow injuries.

**Conclusion:**

Knowledge of the intricate anatomy around the elbow joint is essential to successfully assessing and managing elbow injuries and restoring good function.

## 1 INTRODUCTION

The elbow joint is an important link in the arm between shoulder and hand, and facilitates an arc of hand placement in space under fine control. It is made up of a number of important bony and soft tissue structures that confer stability to the joint and facilitate its kinematics. Importantly for the orthopaedic trauma surgeon, it is a commonly injured joint, and therefore knowledge of its anatomy is essential for undertaking clinical assessment, understanding pathology and planning management.

## 2 OSSEOUS AND ARTICULAR ANATOMY

The elbow is a complex synovial hinge joint involving three separate articulations sharing a common synovial cavity. The ulnohumeral, radiohumeral and proximal radioulnar joints allow combinations of hinge-like flexion and extension of the elbow and pronation and supination of the forearm assisting in hand placement during everyday function.

The wrench-shaped articular surface of the olecranon and the pulley shaped trochlear of the humerus provide bony stability and, together with the strong bands of the medial collateral ligament (MCL) medially and lateral collateral ligament (LCL) laterally, form the primary stabilisers. The radial head, joint capsule and common flexor and extensor origins along side the dynamic muscles crossing the elbow joint form the secondary stabilisers (Table **1**).

Of the primary stabilisers the osseous anatomy provides most of the inherent stability of the elbow joint. The trochlear notch of the ulna surrounds up to 180° of the trochlear proving superior capture [1, 2]. The trochlear is wide in the coronal plane with deep grove in the centre, it forms a 300° arch making it a highly conforming articulation on the ulna [1, 3]. A 30° anterior tilt of the distal humerus comparably matched by the posterior tilt of the trochlear notch works together with the body, tip, anteromedial and lateral facets of the coronoid process to help prevent subluxation of the elbow in flexion and extension (2). Stability at the extremes of motion is further maximised by interlocking of the olecranon process and the coronoid. The radial head acts as an anterior and valgus buttress enhancing elbow stability whilst also contributing to force transmission. Resection of the radial head results in a 30% reduction in valgus restraint which is particularly important where the anterior band of the medial collateral ligament (MCL) is injured as radial head becomes the principle restraint to valgus force (2).

## 3 CAPSULO-LIGAMENTOUS ANATOMY

The MCL and LCL integrate into the joint capsule on the medial and lateral sides respectively to provide stability supporting flexion and extension.

## 4 MEDIAL COLLATERAL LIGAMENT (MCL)

The MCL is triangular in shape and consists of three ligamentous bands, the anterior oblique ligament (AOL), the posterior oblique ligament (POL) and the transverse ligament (TL) also known as Coopers ligament [4]. The AOL Fig. (**1**) originates from the anterior inferior surface of the medial epicondyle of the humerus [5] and inserts into the medial margin of the coronoid process approximately 18mm distal to coronoid tip on the sublime tubercle [6, 7]. The POL also originates from the medial epicondyle and then diverges posteriorly to insert on the medial side of the olecranon with the TL passing between the ulna attachments of the AOL and POL. The AOL is the strongest and most important medial stabilising structure consisting of anterior and posterior bands [8].

The MCL originates posterior to the mid-axial line of the joint, which exerts a cam effect on the elbow joint increasing ligament tension with increasing flexion. The anterior band of the AOL is taut in the first 60° degrees of flexion and the posterior band from 60° to 120° degrees of flexion providing good medial stability independent of joint position.

The POL is fan-shaped thickening of the capsule and forms the floor of the cubital tunnel [9, 10]. The horizontal fibres of the TL extend between the coronoid and the tip of the olecranon. The TL and POL are not thought to significantly contribute to the stability [9] of the elbow joint with POL release having no discernable effect.

The principle restraint to valgus forces acting on the elbow is the AOL with the radial head providing additional stability; where the radial head is excised there is only a limited loss in stability while the AOL is intact. However, gross instability will result where both the radial head is excised and the AOL is released [3, 11]. Following elbow dislocation, the AOL may be compromised, in this situation it is crucial that the radial head remain intact to both counter valgus stress and posterior subluxation of the elbow.

## 5 LATERAL COLLATERAL LIGAMENT (LCL)

The LCL is composed of three parts; the lateral radial collateral ligament (LRCL), the annular ligament (AL) and the lateral ulna collateral ligament (LUCL), this group of ligaments are often referred to as the lateral collateral ligament complex. These three elements provide varus and posterolateral rotatory stability of the elbow and the proximal radioulnar joint. The LUCL Fig. (**2**) principally acts to ensure posterolateral rotatory stability, incompetence of which results in chronic elbow instability [3, 12]. The LUCL crosses the inferior aspect of the radial head with its origin and insertion at the lateral epicondyle of the humerus and the supinator crest of the ulna respectively [8]. The AL stabilizes the proximal radioulnar joint from its insertion and origin at the sigmoid notch of the ulna allowing it to hook round the radial neck. The LRCL originates from the lateral epicondyle of the humerus runs anteriorly to the LUCL and inserts into the AL.

## 6 CAPSULE AND SYNOVIAL MEMBRANE

The tough fibrous membrane of the joint capsule is separated from the synovial membrane by fat pads lying superficial to the radial fossa, coronoid fossa and olecranon fossa. During flexion and extension these fat pads are pulled out of the way by attachments to brachialis anteriorly and triceps brachii posteriorly to allow the accommodation of the adjacent bony processes into their respective fossae.

Anteriorly the joint capsule is attached superiorly to the humerus along the upper margins of the radial and coronoid fossae and to the anterior portion of the medial and lateral epicondyles. Inferiorly it attaches to the coronoid process of the ulna and the annular ligament surrounding the head of the radius. Posteriorly it attaches above the margins of the olecranon fossa superiorly and to the upper margin and sides of the olecranon process and to the annular ligament. In extension, the anterior capsule provides valgus stability [13] whilst the posterior capsule is believed to counter flexion and posteriorly directed forces [14]. In elbow dislocations, the anterior capsule is commonly torn consequently reducing ulnohumeral stability.

## 7 MUSCULOTENDINOUS COMPONENTS

A dynamic component of elbow stability is provided by the muscles acting across it which compress the joint surfaces against one another [8].

Independent of forearm rotation positioning the medial muscle complex consisting of the pronator teres, flexor digitorum superficialis, flexor carpi ulnaris and flexor carpi radialis provides a varus moment resisting valgus forces [15]. Patients presenting with flexor-pronator muscle injuries often also have MCL tears [16] and throwers with chronic MCL weakness frequently have concomitant medial epicondylitis symptoms [17].

Conversely the lateral muscle complex consisting of the extensor digitorum communis, extensor carpi radialis brevis and longus, anconeus and extensor carpi ulnaris acts to counter varus forces by providing a valgus moment. This is of importance in cases where there is an LCL deficiency [14]. It has been demonstrated that even in LCL-deficient elbows there is no significant difference in lateral elbow stability in active flexion whilst supinating the forearm thus supporting muscle activity as an important posterolateral stabilizer of the elbow [18].

## 8 THE “RING” CONCEPT OF ELBOW STABILITY

**Fig. (3):**
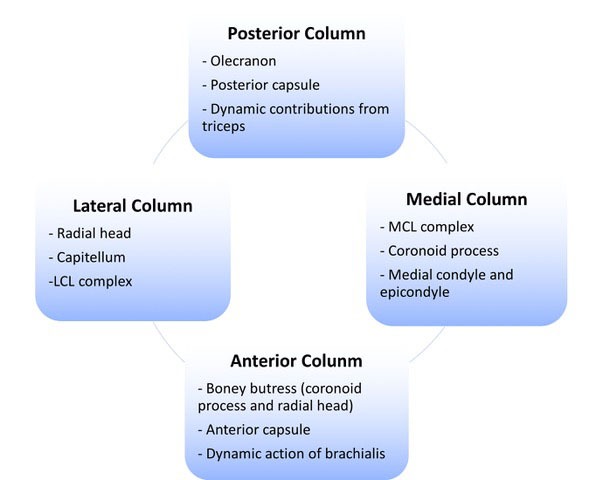
**“**Ring” concept of stability.

The elbow anatomy can be conceptualized as a ‘ring’ structure with medial, lateral, anterior and posterior columns contributing to stability (Fig. **3**) [1]. This concept can be useful when devising management plans for complex instability cases. The anterior column comprises the boney structure of the coronoid process of the ulna acting as a buttress with the attached anterior capsule and dynamic stabilizing forces provided by the action of brachialis. The posterior column is comprised of the olecranon, posterior capsule and a dynamic stabilizing effect of the triceps. The medial column consists of the medial condyle and epicondyle, the coronoid process and the MCL complex. The lateral column is made up by articulation of the radial head on the capitellum with tension forces provided by the LCL complex. The primary stabilizers of both posterolateral rotatory instability and valgus stresses are the AOL and LCL complex with the radial head being an important secondary stabilizer [8].

## 9 CLINICAL ASSESSMENT OF THE ELBOW

Elbow injuries may be considered as either acute or chronic. The initial assessment of acute injuries involves a systematic assessment of the whole patient, as the elbow injury may only be one component of a high-energy multisystem trauma. Relevant aspects of the history in the acute situation include hand dominance, employment history and establishing the mechanism of injury. A history of previous injury to the elbow is useful to identify pre-existing abnormal anatomy. Clinical examination in acute elbow trauma involves identifying open wounds and observing the elbow – the dislocated elbow usually appears deformed with the forearm held in a varus and supinated position. Careful assessment and documentation of the neurological and vascular status of the limb is essential especially if planning to reduce a dislocated elbow. Anteroposterior (AP) and lateral radiographs are the first line investigation with computed tomography (CT) used when dealing with complex fracture-dislocations.

The assessment of chronic injuries requires a more detailed history – key symptoms that the patient may present with include pain, stiffness/loss of motion, instability and loss of normal function related to daily life, work or playing sports. They may also complain of mechanical symptoms such as locking related to loose bodies within the joint. Initial examination involves assessing the carrying angle especially if there has been a previous fracture that may have malunited, and the range-of-motion. If there is loss of elbow motion, it is important to identify end-range pain that may be associated with impingement osteophytes due to post-traumatic osteoarthritis. This may also be associated with symptoms and signs of ulnar nerve compression.

**Fig. (4):**
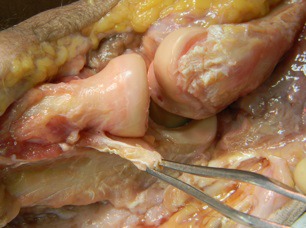
Cadaveric photograph demonstrating posterolateral rotatory instability after transection of the lateral ulnar collateral ligament.

Post-traumatic instability is less common but may manifest as a sensation of apprehension particularly when straightening the elbow for example when rising from a chair or during push-ups. It may rarely present as a recurrent dislocation of the elbow. Assessment involves testing for varus and valgus instability: varus instability is examined with the elbow at 30 degrees flexion and forearm supinated; valgus instability is examined with the elbow at 30 degrees flexion and forearm pronation. Posterolateral rotatory instability is the commonest pattern of recurrent instability and is associated with incompetency of the LUCL [19]. It typically occurs when a valgus load is applied to the elbow in a supinated and extended position thus loading the radiocapitellar joint. This causes the radius and ulna to rotate away from the humerus as a single unit, resulting in the radial head subluxating posterolaterally (Fig. **4**). The instability can be reproduced with the lateral pivot shift test [19]. The patient is placed supine on the examining table with the arm overhead and shoulder externally rotated to stabilise the humerus. The elbow is placed in extension and forearm in full supination while the examiner applies a valgus load which reproduces the instability mechanism. As the elbow is slowly flexed, the radial head is felt to “clunk” back into position as it relocates. It can be difficult to elicit the test successfully in the clinic setting and is therefore often performed under anaesthetic in the operating theatre.

## Figures and Tables

**Fig. (1):**
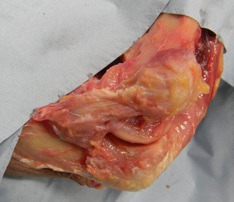
Cadaveric dissection of medial collateral ligament (anterior band).

**Fig. (2):**
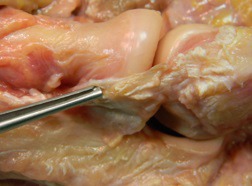
Cadaveric dissection of lateral ulnar collateral ligament.

**Table 1:** Primary and Secondary Stabilisers.

**Primary Stabilisers**	**Secondary Stabilisers**
Olecranon	Radial head
Trochlear	Joint capsule
MCL	Flexor origin
LCL	Extensor origin
	Muscles crossing joint
